# ‘Hiding their troubles’: a qualitative exploration of suicide in Bhutanese refugees in the USA

**DOI:** 10.1017/gmh.2018.34

**Published:** 2019-01-15

**Authors:** F. L. Brown, T. Mishra, R. L. Frounfelker, E. Bhargava, B. Gautam, A. Prasai, T. S. Betancourt

**Affiliations:** 1 War Child Holland, Amsterdam, The Netherlands; 2 Research Program for Children and Global Adversity, Department of Global Health and Population, Harvard T. H. Chan School of Public Health, Boston, MA, USA; 3 SHERPA Research Centre, CIUSS Centre-Ouest de l-ile de Montreal, Montreal, Canada; 4 McGill University, Montreal, Canada; 5 Connection Lab LLC, Somerville, MA, USA; 6 Harvard College, Cambridge, MA, USA; 7 School of Social Work, Boston College, Boston, MA, USA

**Keywords:** Etiology, mental health, qualitative research, refugee populations, suicide

## Abstract

**Background.:**

Suicide is a major global health concern. Bhutanese refugees resettled in the USA are disproportionately affected by suicide, yet little research has been conducted to identify factors contributing to this vulnerability. This study aims to investigate the issue of suicide of Bhutanese refugee communities via an in-depth qualitative, social-ecological approach.

**Methods.:**

Focus groups were conducted with 83 Bhutanese refugees (adults and children), to explore the perceived causes, and risk and protective factors for suicide, at individual, family, community, and societal levels. Audio recordings were translated and transcribed, and inductive thematic analysis conducted.

**Results.:**

Themes identified can be situated across all levels of the social-ecological model. Individual thoughts, feelings, and behaviors are only fully understood when considering past experiences, and stressors at other levels of an individual's social ecology. Shifting dynamics and conflict within the family are pervasive and challenging. Within the community, there is a high prevalence of suicide, yet major barriers to communicating with others about distress and suicidality. At the societal level, difficulties relating to acculturation, citizenship, employment and finances, language, and literacy are influential. Two themes cut across several levels of the ecosystem: loss; and isolation, exclusion, and loneliness.

**Conclusions.:**

This study extends on existing research and highlights the necessity for future intervention models of suicide to move beyond an individual focus, and consider factors at all levels of refugees’ social-ecology. Simply focusing treatment at the individual level is not sufficient. Researchers and practitioners should strive for community-driven, culturally relevant, socio-ecological approaches for prevention and treatment.

Suicide is a major public health concern – over 800 000 suicide deaths occurred globally in 2012, affecting approximately 11.4 in every 100 000 people, and making suicide the 15th most common cause of death, and second leading cause of death in 15–29 year olds (World Health Organization, 2014). Recent data indicate that suicide rates in the USA have increased in the last decade, despite the introduction of national suicide prevention efforts (Cramer & Kapusta, 2017). There is a pertinent need to improve current models of understanding of suicide, in order to enhance prevention and treatment programs, particularly in vulnerable groups.

Refugees from Bhutan constitute one such vulnerable population disproportionately affected by suicide. This ethnic Nepali group was evicted from Bhutan in the late 1980s and lived in refugee camps in eastern Nepal for approximately 20 years (Hagaman *et al*. 2016). Beginning in 2007, over 110 000 Bhutanese refugees from Nepal have been resettled in countries such as the USA (Shrestha, 2015). Recently, the suicide rate among Bhutanese resettled in the USA has been estimated at 24.4 per every 100 000 people, which is alarming when compared with the US population rate of 12.4 (Cochran *et al*. 2013; Hagaman *et al*. 2016). However, reported suicide rates were similarly high for this population while living in Nepal (20.7 per 100 000; Schininà *et al*. 2011).

## Understanding suicide among migrant populations

Models of suicide risk among migrant populations, such as refugees, need to take into consideration a combination of pre- and post-migration experiences and challenges in addition to psychological factors (Vijayakumar & Jotheeswaran, 2010). Quantitative research suggests a relationship between acculturative stressors, social support, and suicidal ideation among immigrant and refugee populations (Hovey & King, 1996; Hovey, 2000; Cho & Haslam, 2010; Akinyemi *et al*. 2015). Of the two quantitative studies that have investigated the elevated suicide risk among Bhutanese refugees, risk factors include symptoms of mental illness, prior exposure to traumatic events, and resettlement-related issues such as language barriers, employment challenges, and family conflict (Ellis *et al*. 2015; Ao *et al*. 2016; Hagaman *et al*. 2016). Thus to understand and address suicidal behavior among resettled refugee populations, it is important to consider factors associated with displacement, resettlement (Miller, 1999; Miller & Rasmussen, 2017), and low rates of health service utilization (Ellis *et al*. 2010; Weine, 2011).

The Interpersonal-Psychological Theory of Suicidal Behavior (IPTS) posits the importance of both social- and individual-level factors in understanding suicide risk. IPTS hypothesizes that two conditions must be met to lead an individual to suicide – both a desire to die and the capability to overcome the innate drive for survival (Joiner, 2007). Two main factors, thwarted belongingness (a feeling of not belonging in important social groups) and perceived burdensomeness (the feeling that one's life is a burden to those around them), contribute to the desire to die (Joiner, 2007; Van Orden *et al*. 2008; Ribeiro & Joiner, 2009). These dimensions are both inherently contextual in that they result from the relationships an individual has with other people and groups, including family, friends, and the larger community in which they live.

IPTS can be paired with an ecological framework to provide a more holistic understanding of suicide. Cramer & Kapusta (2017) propose the Social-Ecological Suicide Prevention Model, which highlights risk and protective factors at the individual, interpersonal/relationships, community, and societal level. A specific social-ecological model, which considers the range and interplay of multi-level risk and protective factors, has the potential to inform the development of more culturally relevant and effective suicide prevention and intervention programs. Such programs would have a scope much broader than the individual, and encompass relationships with families, communities, and the societal context.

## Culture and qualitative inquiry

From an ethnopsychological view, a comprehensive and clinically useful understanding of suicidality should include culturally informed knowledge of perceptions and beliefs (Kohrt *et al*. 2012). For the purposes of this paper, we define culture as ‘a system of shared meanings, institutions, and practices’ (Kirmayer & Ban, 2013). Conceptual models of suicidality still rely largely on Western assumptions and understandings of suicide and mental wellbeing (Cramer & Kapusta, 2017). This is a major shortcoming as the unique historical and contextual experiences of different populations generate culturally specific risk and protective factors for suicide (Chu *et al*. 2018). Additionally, culture influences how people understand and make meaning of suicidal behavior (Staples & Widger, 2012).

Exploring cultural dimensions of suicidal behavior necessitates moving beyond quantitative research methods and utilizing qualitative modes of inquiry to more actively ‘engage’ with suicidality from the point of view of people embedded within the culture (Staples & Widger, 2012). There is a rich body of ethnographic work that has examined the interplay between culture and suicidal behavior among different subpopulations (see for instance Kral, 2012; Gulbas & Zayas, 2015; Straight *et al*. 2015) Unfortunately, to date there has been limited qualitative research aiming to understand and prevent suicide among resettled Bhutanese. In a study of 14 psychological autopsies with family members of individuals who had died by suicide, Hagaman *et al.* (2016) found that disappointment with current unemployment, lack of post-resettlement services and social support, and frustrations with separation from family were contributing factors. These are important findings; however, this study focused on identifying concrete risk factors, as opposed to building a culturally informed model of suicidality in the Bhutanese community. Additionally, qualitative research on this topic that includes the perspective of a wider range of community members is essential, since this has the potential to provide a deep and nuanced understanding of suicide from a broader perspective (Maxwell, 2013).

The World Health Organization recommends selective suicide prevention for vulnerable groups, including those affected by conflict or disaster, and refugees and migrants (World Health Organization, 2014). Yet in order to achieve meaningful impact, it is vital that such interventions are accessible, culturally informed, address relevant needs, and effectively engage community members.

## Current study

The aim of this study is therefore to explore the understandings of suicide of Bhutanese refugee communities in Massachusetts, through inductive thematic analysis of focus group data. A qualitative approach was taken to allow in-depth exploration of this complex issue to inform further research and programming, and a social-ecological model was applied during analysis given the emerging awareness of the importance of considering the multiple levels of influence. The primary research question was:

‘How do Bhutanese refugee community members understand suicide and the perceived causes, and risk and protective factors for suicidality, at the individual, family, community, and societal levels?’

## Methods

### Participants and design

Study participants were 83 resettled Bhutanese living in New England, USA. Eligible youth were aged between 10 and 17 and first-generation resettled refugees born in either Bhutan or Nepal. Eligible adults were a parent, or recommended by community members as someone knowledgeable about challenges facing Bhutanese refugees.

Focus groups were conducted across two phases (see Table 1). In phase 1 (2014), 12 focus groups were conducted: three adult male groups; three adult female groups; three youth male groups; and youth females (three groups). These focus groups explored mental health generally, and this analysis particularly focused on data from one specific question on suicide (or ‘*aatmahatya*’ in Nepali). Focus groups were on average 60–90 min in duration, and time spent on discussion of suicide specifically varied.

**Table 1.** Demographic information of Bhutanese refugee focus group participants

Age group	Gender	Number of groups	Number of participants	Average age [range]	Average number of years in US [range]
Adult	Female	4	25	32 [21–49]	2.9 [0.25–7]
Adult	Male	4	22	39 [22–57]	3.6 [0.3–8]
Youth	Female	3	20	14 [10–17]	2.7 [0.75–5]
Youth	Male	3	16	13 [10–17]	2.5 [0.75–6]

In phase 2 (2016), two focus groups were conducted specifically on the topic of suicide: one adult male group (64 min long) and one adult female group (104 min long). Researchers obtained approval for phase 1 from the Institutional Review Board of the Harvard T.H. Chan School of Public Health (Protocol 15680) and phase 2 was considered exempt from review from the same board (Protocol: IRB16-0600).

### Procedure

These data were collected as part of a larger community-based participatory research partnership between the Harvard T.H. Chan School of Public Health and Bhutanese communities in Greater Boston and Western Massachusetts (Betancourt *et al*. 2015). Phase 2 was also conducted in collaboration with the Greater Boston Coalition for Suicide Prevention as part of a broader project investigating suicide among vulnerable cultural groups. The study team worked in conjunction with Bhutanese research assistants (RAs) and community leaders to identify eligible individuals for participation. Snowball-sampling methods were used to recruit participants for each of the four categories. Adults provided verbal informed consent, and verbal parental consent and youth assent were obtained for youth. All team members were trained in identifying signs of distress in participants, and in following safety plans to identify and address risk of harm cases, including suicidality.

For phase 1, a semi-structured interview guide used in previous studies with refugees was modified for use with resettled Bhutanese (Frounfelker *et al*. 2017). Interviewers began with an open question asking participants for their views on why high rates of suicide were being seen in the communities, and then probed further to elicit specific risk factors, warning, and causes for suicide. The interview guide was developed in English and then translated into Nepali by Bhutanese RAs with extensive experience working with the project, and familiarity with the terminology used within the project.

A semi-structured interview guide for phase 2 was developed in English, incorporating information from phase 1 to further explore seemingly relevant topics, and focusing solely on suicide. It was translated to Nepali by the Bhutanese author facilitating the focus groups (TM). In both cases, translations were reviewed by other bilingual project team members to ensure accuracy.

Focus groups were conducted in Nepali. In most cases, the RA was gender-matched to participants, except in the case of the phase 2 female adult group, in which a male RA conducted the group, due to scheduling. All focus groups were audio-recorded and transcribed directly into English by a Nepali-speaking RA (AP), and transcripts were checked for accuracy and completeness by TM before analysis. All participants attending the groups participated in discussions; however, there were two participants that did not attend the scheduled groups due to family vacation.

### Data analysis

Data analysis was conducted via inductive thematic analysis (Maxwell, 2013). Initial familiarization with the data was achieved by repeated readings of the transcripts by two authors (FB and TM), one of whom is a Bhutanese refugee himself (TM). Next, all transcripts were ‘open coded’ by FB to identify and label all segments of data perceived relevant to the research questions. Through discussions with TM, a code book was developed consisting of categories linking back to our central research questions. Major categories were assigned labels (codes) and definitions. All transcripts were coded by FB using MaxQDA software, and the code book iteratively revised based on data and discussions with TM. Codes were populated with data in the form of direct quotes from transcripts, and then grouped into key sub-themes and themes. Themes were reviewed for consistency based on the coded extracts for each theme, as well as the applicability and comprehensiveness of the set of themes for the full data set which was discussed and validated with TM. Names and definitions were developed for each theme and extracts of data most illustrative of the themes were selected for display in the present manuscript. Additional authors with experience in conducting qualitative research on suicide in various cultural groups (EB) and with experience with Bhutanese refugee communities (BG; RF) reviewed the overall themes and findings and provided further input. No major new ideas emerged during analysis of the final transcripts, suggesting that data saturation was achieved.

It is important to note that focus group guides did not specifically probe on levels of the social-ecological model, and the importance of using this model to understand suicide emerged on reflection after conducting the groups, and during early stages of data analysis.

## Results

**Fig. 1.**
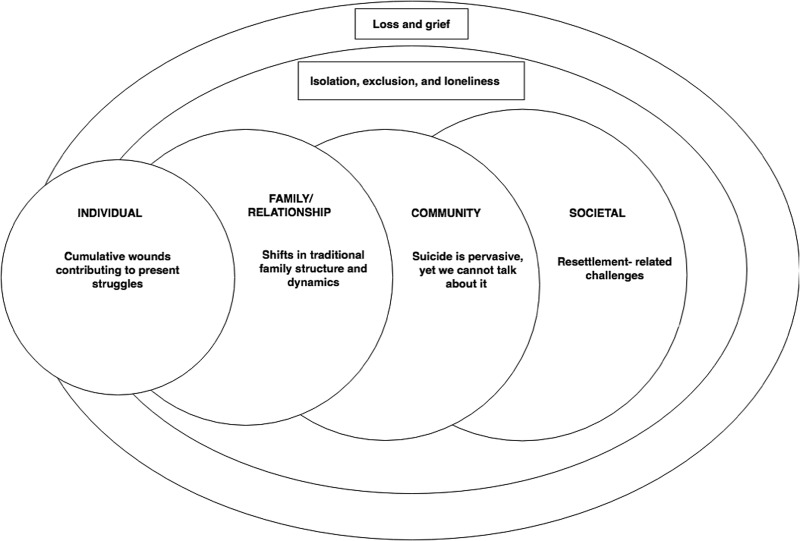
Themes related to Bhutanese suicidality, situated in a social-ecological model.

There were six overarching themes related to understanding perceived causes, and risk and protective factors for suicidality in the Bhutanese refugee communities. These themes both resided in, and cut across, individual, relational, community, and societal levels (see Fig. 1). In the presentation of themes below, difficulties experienced at the individual level are presented last, as it was perceived that these difficulties were best understood after presentation of themes at other levels.

### Societal level

#### Theme 1: resettlement-related challenges

Participants consistently identified multiple resettlement and acculturation-related challenges influencing mental health and wellbeing. These difficulties contributed to a sense of ‘*cultural shock*’, which was exacerbated by needing to adjust quickly to many new things in all aspects of life.

Language and literacy barriers impacted individuals on several levels. An adult male described how they impact social connectedness: ‘*They* [Bhutanese refugees in a workplace] *can't speak, they don't know how to, and you grunt this way and that way but you can't make friends. And what happens then, is they think that what would be easier is just to hang themselves and die.*’ Language barriers also impact help-seeking, due to the requirement for interpreters. There were issues reported relating to quality and understandability of interpreters, or lack of trust in confidentiality, given that interpreters are often from the same community. One adult female noted, ‘*And now just to talk to someone else outside, there's a language barrier. You have to get an interpreter. The interpretation isn't face-to-face in mental health and when they give you a phone interpreter, there is no clarification.*’

Employment, study, and financial challenges also contribute significantly to distress, with immense pressure felt around providing for oneself and family, and a discomfort with loans and repayments, which were reported as not culturally common. A child discussed this relationship between economic strain and suicidality, ‘*The ones who want to do it* [*suicide*]*… they're the ones who don't have work, who don't understand anything, problems at home, not being able to pay money, it happens to people like that.*’ There was consistent mention of working long hours to survive financially, and challenges in finding work, or meaningful work. Younger generations often need to work rather than study, yet this limits employment opportunities available to them. This can lead to feelings of hopelessness, as one woman explained:

‘*It's like: ‘I go to work, I come back, I eat and I sleep. I don't have a life, the rest of my life this way, I have to pay for these electrical bills, my parents are struggling, things in my household aren't going well. I am not able to meet up with any friends and I can't say anything. If the rest of my life is going to go this way…’ Just thinking of how their life used to be, just thinking of this can make someone do suicide, I think.*’

Finally, uncertainty and stresses about obtaining citizenship, and therefore being able to continue to receive government benefits after the 7-year period allowed for recently resettled refugees, were frequently cited as contributing factors for suicidality, particularly among older adults. One adult male explained:

‘*They have* [*Social security insurance*] [*…*]. *Tomorrow, when it stops coming… they can be a burden to the children… this is something that's giving the parents a lot of tension and suicidal rates in these people can increase. Because not everyone can raise their parents here like they did back there, they don't have time,* [*….*] *And in the elders, there's a lot of tension…*[*….*] *how can they easily get citizenship out? If you could do advocacy on this, that would help prevent suicide*’

### Community level

#### Theme 2: suicide is pervasive, yet we cannot talk about it

In the focus groups, it was emphasized that suicide is common in these communities, with a history beginning in Bhutan, occurring in refugee camps in Nepal, and continuing throughout resettlement in the USA. Yet, there is a silence around mental health and suicide among community members. This unified theme was noted given the striking paradox evident, and since the essence of the theme was related to the discrepancy between high prevalence, yet limited discussion. Suicide was portrayed as strikingly normalized in the communities, particularly when the method was hanging: A man stated, ‘*If they died with a gunshot, there would be a bigger deal because there is no tradition about that. If they hung themselves and died, it's* [*the conversation around event*] *over.*’ There was a fear that knowledge of other suicides would increase the risk for further suicides – one female participant described the frequent suicides as a ‘*wave*’. This risk could be transmitted by children being impressionable and mimicking what they observed: ‘*When in the camp, seeing people being hung and die, they* [*children*] *see that and when they see that, they try it*’ [adult female]. Additionally, individuals might consider the life experiences and reasons behind an individual's suicide, compared them to their own, and decided that suicide was a viable option. One adult female articulated:

‘*You don't need to see anyone doing suicide… how this affects the mind is that they think… ‘yeah they did it… and then what happened? Oh in Bhutan, they had a car… they had this… and look at them now, they're in the camp… they have nothing…not even wood for the fire.’ That happens and they think they are ruined. And another… like ‘I had an orange field, I had this, I had this, I left everything. Woah…maybe these things are going to affect me too’. And it happens and happens and happens and that thing DOES affect them.*’

Although knowledge of suicides was repeatedly common, participants identified an inability or unwillingness to further discuss mental health issues within the community. This was within family and close friendship groups, and was possibly due to less awareness, different cultural concepts of suffering and support, or perceived stigma around such difficulties:

‘*No one talks about depression, that doesn't even come up. People from morning to night are sitting annoyed and sad, but the act of asking someone what happened… that doesn't even come up. There is less awareness about mental health so there are many crises in a new place. There are so many crises that we can't solve*’. (Adult male)

Within the data, there was mention of a common belief within the community that you should not talk about personal or family issues outside of the family, which further increases the silence about mental health concerns and lack of access to informal support. A commonly referenced fear was around speaking to health professionals, and in particular to interpreters who are often from the community, due to potential lack of confidentiality and associated fear of stigma, and this acts as a further barrier to seeking professional support. In addition, cultural differences in engagement with health care and the way questions about mental health are asked, contribute to people ‘hiding their troubles’.

‘*in America, when they come to ask you, people share all their feelings with their therapist. In ours….because of stigma, they'll think this person is crazy, they're mad [….] In our people, we have been unable to clear that doubt and up until people don't trust, they won't say anything, and because they don't say anything, no one will know and all of a sudden, they will explode*’ (Adult male)

### Relationship level

#### Theme 3: shifts in traditional family structure and dynamics

Participants reported changes in relationships and power dynamics between parents and children, and between partners. A role-reversal was common between parents and children as parents came to rely on children for assistance with language and understanding US systems, leading to more responsibility and pressure for children, and a loss of role and a sense of dependency for parents. Given financial challenges, role-shifts in families related to work are common, whereby children feel pressure to gain employment rather than study in order to help their family, and women enter the workforce more frequently. A young adult female talked about the challenges balancing work outside the home, with criticism for not fulfilling traditional family duties:

‘*We have to work. [….]. If we go to work, there is no one to take care of our mother-in-law and if we stay at home, there's the ‘what's the daughter-in-law doing just at home taking care of her mother in law… she should be at work’ concept.*’

These challenges frequently lead to a lack of understanding, lack of communication, and conflict between generations. Specific to parent–child relationships, one adult female noted, ‘*Children get up early and go to school and when they come back from school to home, they're in a different environment, in a school a different environment. Because of that…what I see now is that actually the conflicts between the children and the mothers and fathers is a really big thing.*’ This generational divide was reportedly exacerbated by the faster trajectory of acculturation for younger generations, through their school and peer networks. An adult female stated, ‘*if the one walking in front doesn't wait for the one walking behind, even running can't make them catch up. I'm trying to say…the children are going up front and the parents are being left behind.*’ These conflicts and lack of family cohesion could further exacerbate distress for individuals. A different woman explained, ‘*Whatever is not in our culture, it's hard for parents to accept…They think that and try to accept their children but they can't… like the mentality they've been building for 35 or 25 years, parents… it's not going to go away within 2 years.*’

For children and younger generations, they also feel the need to maintain a dual cultural identity, whereby they need to adjust quickly to the American culture outside the home (in work, school, society), yet maintain Bhutanese traditions and customs at home. One woman reflected:

‘*We are living two lives at once…. Throughout the day, we are American, we go outside, make money, work, and then come home and we are Nepali and make our parents happy. So the young generation has the worries of their parents. So the young generation also has as much problems… there are things and people we're trying to make happy. Life is very hard.*’

### Cross-cutting themes

#### Theme 4: loss and grief

A theme that cut across ecological levels was a broad sense of loss and the associated accompanying grief. Firstly, material losses were identified among older generations coming from Bhutan or Nepal in terms of ‘*land, food, fields, riches* [wealth]*…*’ [adult male]. There is also a loss of culture and community, due to: lack of spaces and time to come together; an inability to conduct certain cultural rituals without suitable venues and understanding of neighbors; and competing financial pressures and acculturation processes. One adult male explained, ‘*If we wanted to say, ‘let's meet and do bhajan or eat or meet’, we don't have a place. Because we can't afford halls.* [*town*] *is a crowded area and we tried. We tried to afford rent. And when there is no social activity, people have to stay at home.*’ There was also a sense of less tangible loss related to a loss of dignity, ‘*prestige*’, and respect from others. This was particularly salient among older adults, and occurred both within the family system and larger community. One adult female reflected on this, ‘*They* [*older adults*] *think, ‘When I was there, so many people followed and respected me and here, not even my grandchildren respect me… So that means… how wasted have I become?*’ This results in a loss of hope and sense of purpose in life for older adults who feel left behind by their extended family. One woman noted that if a sense of purpose was linked to children, and children were settling into American life and taking care of the family, this could lead to thoughts such as ‘*Like what could I even be doing now… what else do I have left to do but die?*’.

Adults often feel a loss of independence and increased reliance on others – whether it is children for financial or practical support, or interpreters for seeking help from health professional. One man explained the relationship between language barriers and this feeling of loss as:

‘*Even the smart people become dumb here. Because of language…they are forced to be disabled. And the disability makes people nauseous. Their voice doesn't work. The things they worked so hard to bring here, isn't here. And they don't have the satisfaction of making and bringing something with their own hands… And they become dependent. And when they become dependent, there's a bruise to their prestige… And they become sad and do suicide.*’

A further sense of loss was related to the unmet ‘American dream’. When preparing and arriving in the USA, there was the sense that life would improve and a certain standard of living would be enjoyed. However, many Bhutanese refugees experience a vast expectation–reality gap, leading to the loss of an imagined better future, which causes distress.

‘*People coming to the US, they did not come here to carry cartons. From the beginning, they thought that they would study and become big people. What they think about Americans is that when Americans go to Nepal, they spend so much money. Right?… they come to America with that dream. Come here and study and be a big person and when they're a big person, go back to Nepal on vacation and everyone will think of them highly. But they don't realize the reality here… coming here, another reality is that they can study, but if they're 19, they can't study. Go and work! Did you get a job? No. The expectations they have can't be met.*’ (Adult male)

#### Theme 5: isolation, exclusion, and loneliness

These challenges across different ecological levels lead to feelings of isolation, exclusion, and loneliness. In focus groups, there was a sense of individuals often feeling alone and lacking social connections; this could be due to relationship disruptions during resettlement, and trouble connecting with the new community.

‘*And coming here, they have to sit alone and because of that […] they have grave thoughts, like they can't talk like they want, they can't do like they want, they don't understand… because of that, children… 16 or 17 year olds, they do suicide, I think*.’ (Adult male)

For children and youth, commonly cited issues were bullying and teasing at school and online, or fights with friends, creating a sense of isolation and exclusion. As one female youth explained: ‘*the foreign people get bullied more because they don't know the language and sometimes they think we don't understand what they are saying and they keep going about it*.’

Environmental factors contribute to these feelings, as communities are quite separated, and people are not connected to neighbors. One adult man stated: ‘*We hear we have neighbours but where are they?*’. Participants cited that in Bhutan and Nepal, it was common to leave the door to your home open, yet in the USA it must be kept closed, thus leading to a further sense of confinement and isolation. For older generations, there was even more sense of being confined to home due to weather, safety concerns, or language barriers.

‘*To say it straight,… when we stay inside, we don't close the doors. We need to keep the door open, we came from the place like that. Here you have to close the door. In case someone comes from outside, we have to close the door. That is, for our parents, it's a big thing. It affects our culture* [*….*] *What kind of place is this… for the parents?*’ (Adult female)

In these conditions, loneliness, boredom, and lack of connection is common and described as unbearable. One adult male described the need for engaging activities and connections for isolated older people: ‘*What to do? There's nothing. And with no options, there are definitely thoughts… when the person gets to the destination, if they have no thoughts about where to go… now… a new program, if you don't put a new program in, my mom doesn't have another destination.*’

### Individual level

#### Theme 6: cumulative wounds contributing to present struggles

Participants described multiple traumas and adjustments that individuals within the community had experienced, from forced migration, conditions of adversity in Nepal, and sudden changes and acculturation stressors upon third-country resettlement. This was attributed as a cause of poor mental health and suicidality. There was a sense of accumulation and compounding of traumatic experiences to the point that individuals could no longer cope. For instance, an adult female stated, ‘*When I kept thinking about it, I realized these were the reasons* [*for suicide*]*… in the country this happened, and they had to leave. When they left, this happened. And when they finally came here, and when they couldn't adjust, what should they do? … the wound kept being hurt, it kept going.*’

These past experiences, along with previously described influences from other social-ecological levels, contributed to current thoughts, feelings and behaviors that could be instrumental in suicidal ideation and attempts. These included thinking too much, and in particular thinking negatively about oneself and one's situation. An adult female explained: ‘*There are so many things in your head going on … and there are no places to put their thoughts and thinking about it and thinking about it, they develop depression and because of that, I think they die because of that.*’ Individuals discussed a sense of hopelessness about the future. One female youth described, ‘*like sometimes when you don't think you're good enough for something or somebody or other people…. If you keep trying, and you keep feeling the same thing again and again, sometimes like you just get tired of that like you stop trying, like you don't want to live anymore.*’

Alcohol and drug use, while potentially a coping strategy, conversely was described as leading to further isolation and increased risk of suicide:

‘*People who do drugs or alcohol… the community doesn't think of well of them. He is outcast. And if everyone shuts him out, what does he think of himself?*[*…*] *I'm living, I'm working, who am I working for? There is no one*…’ (Adult male)

## Discussion

This study investigated the way in which Bhutanese refugee community members understand the perceived causes and risk factors for suicidality at individual, family, community, and societal levels. Themes identified can be situated within different levels of the social-ecological model, as well as cutting across multiple levels. At the individual level, current thoughts, feelings, and behaviors can only be fully understood when considering past experiences, and stressors at other levels of the ecology. Shifting dynamics and increased conflict within the family were pervasive and challenging, with children feeling pressure to take care of older generations, and parents feeling a loss of status in the family. At the community level, the high prevalence of suicide was noted, yet conversely there were barriers to communicating within families and communities about mental health and suicide. At the societal level, difficulties relating to acculturation, citizenship, employment and finances, and language and literacy were influential.

Findings highlighted the importance of considering multiple levels of the social ecology. One example of this was the experience of a large amount of pressure felt to obtain citizenship within the required 7-year period, in order to continue receiving government benefits. Individuals with certain mental health conditions may get a waiver for taking citizenship tests. Yet there is poor access to mental health care services where such diagnoses may be made, among arguably the most vulnerable Bhutanese refugees, due to factors such as language barriers and perceived stigma around certain kinds of psychological distress. Thus individuals do not receive diagnoses for conditions that may exempt them from citizenship tests, and furthermore do not receive treatment for increased distress and worry felt as a result of this pressure. If such distress was not considered in the broader context of these challenges, several important avenues for suicide prevention would be missed.

Study findings provide support for the relevance of the Interpersonal-Psychological Theory of Suicide Behavior when understanding suicide among resettled refugees. In particular, the dimension of thwarted belonging seems particularly salient among older Bhutanese. Isolation, exclusion, and loneliness was felt at the individual level, yet was related to changes in family and community structures. Financial pressure necessitated family members work long hours outside the home, and led to concerns about older generations turning to suicide as a way to end suffering. This finding is consistent with existing research on the needs of older Bhutanese and risk factors for adverse mental health outcomes among this subgroup (Gautam *et al*. 2018; Kim *et al*. 2017), and lends support to an ecological framework to understand how these stressors may contribute to overall risk of suicidal behavior in the community.

Study findings provide more in-depth information on the relationship between culture and suicide behavior among Bhutanese. More specifically, the qualitative data allow us to unpack the broad construct of ‘acculturation stress’ that has been defined as a risk factor for suicide in quantitative research. Study participants articulate understanding suicidality as a behavioral response to cultural loss. This loss manifested itself differently depending upon age and gender. Adult men and women experienced a shift in gender roles and familial expectations that could become untenable; youth grappled with retaining a Bhutanese identity but fitting in with their new, American culture; older adults faced losing the prestige and status that had formerly been conferred on them and had been embedded within cultural tradition. Still other participants discussed a loss of not being able to literally ‘engage’ in their culture, such as participating in religious ceremonies and practices. The relationship between cultural loss and suicide has been explored in other marginalized populations, notably Alaskan Natives and the Inuit in arctic Canada (Wexler, 2006; Kral, 2012, 2013), and should be integrated into models of suicidality among displaced populations. Literature in aboriginal populations has highlighted the phenomena of clustered suicides as a manifestation of attempting to ‘belong’ in the midst of not feeling you belong (Niezen, 2017). Puzzlingly, many of the difficulties facing Bhutanese refugees are common across other refugee groups moving to the USA, yet higher suicide rates are extant in this population. Therefore, further exploration of a possible ‘cultural tradition’ of suicide among Bhutanese refugees, or other reasons for possible ‘suicide contagion’, may be particularly useful.

### Implications for conceptualizing suicide

These findings point to the value of taking a broad social-ecological approach when understanding suicide among Bhutanese refugees. While many theories of suicide tend to focus at the individual and/or family level (Cramer & Kapusta, 2017) we found that many of the perceived risk factors for suicide were not directly related to individual emotional or psychological suffering. Instead, root causes were often perceived to be external factors at different levels of the ecology. Community- and societal-level factors appear to be pertinent when considering suicide in this population, and cultural factors appear to dramatically interrupt family dynamics, relationships, and support. This is in line with qualitative findings from Chase & Sapkota (2017) considering help-seeking among Bhutanese refugees, and quantitative studies in non-Western countries including Nepal (Jordans *et al*. 2018; Thapaliya *et al*. 2018). Therefore, potentially important risk factors such as poverty, unemployment, disrupted family, and social support systems, and educational challenges for Bhutanese refugees resettled in the USA must be considered.

The value of an ecological approach has also been underscored when considering the refugee resettlement experience more broadly. Miller (1999) highlights the significant distress that arises from ongoing daily stressors, including social isolation, loss of social and occupational roles and the corresponding loss of meaningful activity, environmental mastery, and material and financial resources. Each of these factors was represented in the key themes from this study. While a traditional war-exposure model of refugee distress will focus on historical events and traumas as key contributing factors and foci of intervention, Miller & Rasmussen (2017) highlight the need to pay attention and intervene with displacement-related stressors such as poverty, unemployment, social isolation, and family conflict.

### Implications for mental health treatment and suicide prevention

These findings highlight the importance of holistic mental health treatment and suicide prevention programs that move beyond traditional medication and individual psychotherapies and look at relational processes and the social environments that individuals are situated within. Promoting mental health among individuals may assist in preventing suicide; however, it is unlikely to be sufficient given the complex interplay of factors at other levels of the social ecology. Further, it is important to recognize that interventions that focus on perceived ecological causes of distress, via providing pragmatic support without directly targeting individual emotional suffering (in the way that typical psychological interventions might), may be valid and effective approaches for both mental health promotion and suicide prevention. For example, Hagaman *et al.* (2016) recommended that interventions with Bhutanese refugees include training the community in communication strategies to address suicide, engaging newly resettled families immediately into social and educational opportunities, and enhancing and improving resettlement services and support. Among elderly Bhutanese refugees in the USA, the two most promising programs suggested for increasing psychological adjustment were bilingual citizenship classes (to assist them in passing the citizenship test) and social activities (to encourage social interaction and build social capital) (Kim *et al*. 2017).

Chase & Sapkota (2017) highlight the importance of community support, the informal care sector, and non-stigmatizing approaches to intervention for psychological distress, including proactively identifying and addressing observed changes in behavior and experienced stressors. While not contradicting the importance of family and community support, the findings from our study suggest that the communities in our sample feel somewhat limited in their ability to utilize and provide this support to one another – for example, due to the lack of discussion about it in the community, the cultural divide, and lack of understanding between different generations within families, the loss of community structures and processes to support such cohesion and support, and the immense pressures facing individuals which leave limited time to engage with family and community. It is likely that non-stigmatizing interventions to promote these systems will be helpful.

Promisingly, there is preliminary evidence that targeting daily stressors and resettlement challenges may improve wellbeing among refugee populations in general (for an overview see Miller & Rasmussen, 2017), and specifically in Bhutanese refugees. For instance, Mitschke *et al.* (2013) found that a financial literacy program was associated with improvements in psychological wellbeing among Bhutanese refugee women resettled in the USA, and Gerber *et al*. (2017) found potential benefits of community gardening for increasing social support. Based on our findings, it is likely that a range of programs to promote positive mental health and prevent suicide will be most effective. This might include individual counseling or psychotherapy operating at the same time, or in an integrated manner, with: strategies to increase access to appropriate care, family-focused interventions, school-based interventions including targeting bullying, community spaces and provision of cultural and religious activities, awareness programs, gatekeeper training, practical support throughout the resettlement process, and advocacy for policy changes and funding improvements. Further, investment could be made in capacity development trainings at community leadership level to empower and leverage them to search for available resources at local, state, and federal level to address the social, cultural, and educational needs of their community.

### Limitations

There are several limitations that should be noted. Only adult participants in phase 2 took part in a full interview on suicide (*n*  =  11); the remaining participants only answered one question on suicide, as part of a larger interview. Due to the small number of focus groups and the lesser contribution of children on the specific topic of suicide, findings are potentially limited in terms of generalizability to the wider Bhutanese refugee population, and largely reflect adult perceptions and understandings. Given time constraints in the interviews, we did not ask respondents to specifically distinguish between risk factors for different age groups. Given the different acculturation trajectories and challenges, it is possible that a comparative study would identify important differences between age groups.

Since the study utilized focus group methodology rather than individual interviews, and included participants knowledgeable about the challenges facing Bhutanese refugees, but not specifically those experiencing mental health difficulties themselves, the results only capture the perspectives of others on individual difficulties rather than fully capturing the direct experience of individuals. Further, the adult female focus group was conducted by a male. While the judgment was made that this would be culturally appropriate in this instance, there is the possibility that the gender difference between interviewer and interviewees may have influenced the results such that participants were less comfortable to disclose their own experiences.

There are inherent possibilities for bias and subjectivity in qualitative research. However, FB and TM were both trained in Maxwell's qualitative research methodology (2013), which emphasizes researchers’ prior experiences, interests, beliefs, culture, and expectations and acknowledges the influence this can have on analysis. Thus FB and TM discussed issues of reflexivity throughout analysis. It was acknowledged that individual factors may be a threat to validity, but may also offer insights.

## Conclusions

This study indicates that causes of suicidality among resettled Bhutanese refugees in the USA are varied, and fall across all ecological levels. Beyond past traumas, and current sociodemographic and acculturation difficulties, individuals experience dramatic shifts in family relationships and roles, and feelings of loss and isolation, which contribute to perceived pressure and feelings of uncertainty and hopelessness about the future. This occurs in a context where expectations of the ‘American dream’ have not been met, and it is difficult to express concerns to family, friends, or professionals. Models of understanding and intervening for suicide must move beyond an individual focus, and consider innovative intervention at all levels of the ecology. Failure to develop holistic, culturally relevant, and ecological programs for intervention and prevention will hinder efforts to reduce the prevalence of suicide in this group.
